# Respiratory protection for healthcare workers caring for COVID-19 patients

**DOI:** 10.1017/ice.2020.175

**Published:** 2020-04-23

**Authors:** Leonard A. Mermel

**Affiliations:** ^1^Department of Medicine, Warren Alpert Medical School of Brown University, Providence, Rhode Island; ^2^Division of Infectious Diseases, Rhode Island Hospital, Providence, Rhode Island; ^3^Department of Epidemiology and Infection Control, Rhode Island Hospital

*To the Editor—*The infection prevention practices that should be followed when caring for COVID-19 patients remain uncertain. Preventative strategies can be categorized as (1) source control of respiratory particles from infected patients and (2) respiratory personal protective equipment worn by healthcare providers.

## Source control of infected patients

Johnson et al^1^ studied source control in influenza-infected patients while coughing. Although an average of 50,000 viral RNA copies/mL were detected 20 cm away from 7 of 9 unmasked, coughing patients, no influenza viral RNA was detected 20 cm from coughing patients wearing either a surgical mask or an N95 respirator.

Milton et al^2^ reported a 2.8-fold reduction in viral RNA in respiratory particles ≤5 µm and a 27-fold reduction in respiratory particles >5 µm when influenza-infected participants wore surgical masks. Although the investigators detected influenza viral RNA in respiratory particles ≤5 µm in 78% of influenza-infected individuals, viable virus was detected in only 5% of these individuals (ie, 2 of 37 individuals who were unmasked and 2 who were masked). This seminal finding demonstrates that detection of viral RNA may be of limited clinical utility when assessing risk of respiratory viral transmission unless it is accompanied by an assessment of viral viability.

Leung et al^3^ assessed viral shedding in individuals infected with various human respiratory viruses when they were coughing, with and without wearing a surgical mask. They were unable to detect human coronavirus RNA in respiratory particles ≤5 µm or >5 µm in the surrounding air of infected patients wearing a surgical mask. Influenza virus and rhinovirus RNA were detected in 4% and 22% of respiratory particles >5 µm from masked patients, respectively, as well as 22% and 38% of respiratory particles ≤5 µm from masked patients, respectively. Notably, viral RNA was detected in a small number of participants who did not cough during the 30-minute exhaled breath collection, suggesting the risk of respiratory viral transmission from asymptomatic individuals.

Bae et al^4^ assessed source control in COVID-19 patients with detection of viral RNA ~20 cm from each patient’s face while they were coughing and wearing a surgical mask or cotton mask. They found no significant reduction in viral RNA detection when participants wore either of these 2 masks. Curiously, the authors were unable to detect viral RNA from the inner surface of the surgical mask or cotton mask in 3 of the 4 participants despite finding viral RNA on the outer surface of the masks in all 4 participants.

Of the 4 studies noted, 3 did not assess viral viability. The study using viral cultures found viable influenza virus in respiratory particles ≤5 µm of exhaled breath in only 2 of 37 masked patients^2^; both patients had >10,000 viral RNA copies detected over the 30-minute assessment period. Thus, the study assessing viable virus found great utility in reducing viable influenza virus when an infected patient dons a surgical mask.

## HCW PPE

Concern regarding airborne SARS-CoV-2 has arisen based on the detection of SARS-CoV-2 viral RNA in the air of patient rooms. Santarpia et al^5^ detected SARS-CoV-2 RNA in 2 of 3 COVID-19 patient rooms >1.8 m (6 feet) from each patient and in 8 of 12 air samples in the hallway outside patient rooms. Some COVID-19 patients were receiving supplemental oxygen, but the report did not specify whether they were receiving aerosol-generating procedures (eg, nebulized medication) at the time of, or prior to, the assessment of viral RNA in air samples. Most importantly, utilizing 2 different methods, viable virus was not detected in air samples. A low concentration of viral RNA in air samples may have made viable viral detection difficult. These results suggest that finding viral RNA in the air without viable virus, at least at the lower level of detection utilized, is of uncertain clinical significance. In contradistinction to results from patient rooms, Van Dorenmalen et al^6^ created aerosols using a 3-jet collision nebulizer to aerosolize SARS-CoV-2 into the air at a concentration similar to that found in respiratory tract secretions and found the median estimated half-life of viable virus to be 1.1 hours (95% credible interval, 0.6–2.6 hours).

Guo et al^7^ detected SARS-CoV-2 RNA in air samples in intensive care unit (ICU) rooms of COVID-19 patients, non–ICU rooms of COVID-19 patients, and near air outlets. Although viral RNA was detected in air samples, the investigators were unable to detect any viral RNA on the face masks of healthcare workers in ICU and non-ICU settings. Similarly, Ye et al^8^ detected SARS-CoV-2 RNA in only 1 of 58 (1.7%) of healthcare worker’s face shields or other devices used for eye protection.

In a meta-analysis of published studies involving 5,549 healthcare workers randomized to wearing a surgical mask or N95 respirator during non–aerosol-generating patient care activities, Alhazzani et al^9^ found no significant difference in laboratory-confirmed respiratory viral infection, influenza like illness, or clinically defined respiratory viral infection. However, the odds ratio increased from 1.06 for laboratory-confirmed infection to 1.31 for influenza-like illness and to 1.49 for clinically defined respiratory infection. These results suggest the possibility that N95 respirators may have a more protective effect. Again, none of these differences were statistically significant in the analysis of 5,549 healthcare workers. The analysis was not stratified by use of source control, such as a surgical mask, worn by patients when in close contact with healthcare workers. One of the studies^10^ in the meta-analysis found no significant difference in risk of human coronavirus infection between the 2 groups. In a retrospective study, Seto et al^11^ found that wearing a surgical mask or N95 respirator significantly reduced risk among healthcare workers caring for COVID-19 patients. Ng et al^12^ found that none of 35 healthcare workers who had worn a surgical mask when caring for COVID-19 patients became infected, including patients receiving aerosol-generating procedures such as endotracheal intubation.

The aforementioned studies suggest that donning a surgical mask offers protection to healthcare workers when caring for patients infected with various respiratory viruses. With 1 exception, these studies focused on patients who were not undergoing aerosol-generating respiratory therapy or procedures. A retrospective study^11^ found that wearing a surgical mask or an N95 respirator significantly reduced risk among healthcare workers caring for COVID-19 patients and an observational study^12^ found that wearing a surgical mask caring for COVID-19 patients did not lead to any infections among 35 healthcare workers. However, a meta-analysis revealed a nonsignificant increased risk of influenza-like illness and clinically defined respiratory viral infection among healthcare workers wearing surgical masks compared to an N95 respirator without concomitant source control assessment. Although viable SARS-CoV-2 has been shown to have a half-life of 1.1 hours in experiments using a 3-jet collision nebulizer to aerosolize the virus,^6^ viable virus was not detected in patient rooms in another study.^5^

Based on the totality of data, the utility of source control of infected patients using a surgical mask while healthcare workers wear a surgical mask and eye protection,^13^ particularly with a face shield, should afford a high degree of protection when caring for COVID-19 patients who are not undergoing an aerosol-generating procedure. For such procedures, an N95 respirator is recommended for respiratory protection; a powered, air-purifying respirator may afford a higher degree of protection in such circumstances.^14^
